# Quantum Random Number Generation Using Nanodiamonds and Nanopillar-Isolated Single NV Centers

**DOI:** 10.3390/nano16070404

**Published:** 2026-03-27

**Authors:** Oskars Rudzitis, Reinis Lazda, Valts Krumins, Heinrihs Meilerts, Mona Jani, Marcis Auzinsh

**Affiliations:** Laser Centre, University of Latvia, Jelgavas Street 3, LV-1004 Riga, Latvia

**Keywords:** quantum random number generation, nitrogen-vacancy centers in diamond, confocal microscopy, single-photon detection, nanodiamonds, nanopillars

## Abstract

Quantum random number generation (QRNG) provides fundamentally unpredictable randomness derived from intrinsic quantum processes. In this work we demonstrate two solid-state, room-temperature QRNG implementations based on nitrogen-vacancy (NV) centers in diamond, i.e., ensemble fluorescence from nanodiamonds and single-photon emission from single NV centers located at the tips of fabricated diamond nanopillars for enhanced light collection efficiency, spatial isolation and minimized crosstalk. We compare entropy rates (above 0.98 bits), statistical performance, and robustness of both approaches in our experimental setup, the results contribute to establishing diamond-based QRNG as a scalable solution for quantum-secure randomness generation.

## 1. Introduction

Random numbers are essential to cryptography, simulation (Monte Carlo [1], protein molecule dynamics [2], and other physical systems that require true randomness), secure communications and quantum key distribution. Classical random number generators are inherently deterministic and rely on algorithmic complexity. In contrast, quantum mechanics provides intrinsic unpredictability. Solid-state QRNG platforms are desirable for room-temperature operation, chip-scale integration and long-term stability.

The oldest QRNG implementations were based on radioactive decay, and they have decreased in popularity due to low bit rate and necessity for radioactive materials [3]. Noise in electronic circuits is another implementation of QRNG, but they suffer from the issue that it is difficult to separate quantum flicker noise from thermal noise and can show memory effects [4]. Most QRNG platforms are based on quantum optics and benefit from the relative affordability of optical devices [5]. In contrast to NV center-based QRNG implementations, a large class of modern QRNG systems relies on internal physical noise sources such as vacuum fluctuations, amplified spontaneous emission, or laser phase noise [6]. These approaches convert intrinsic fluctuations in optical or electronic fields into random numbers and can achieve significantly higher generation rates, ranging from Mbps to Gbps in integrated photonic platforms [7,8,9]. While these approaches provide high throughput, they often rely on indirect entropy sources and require careful modeling and calibration of classical noise contributions, motivating interest in physically well-defined single-photon emitters such as NV centers.

**Figure 1:**
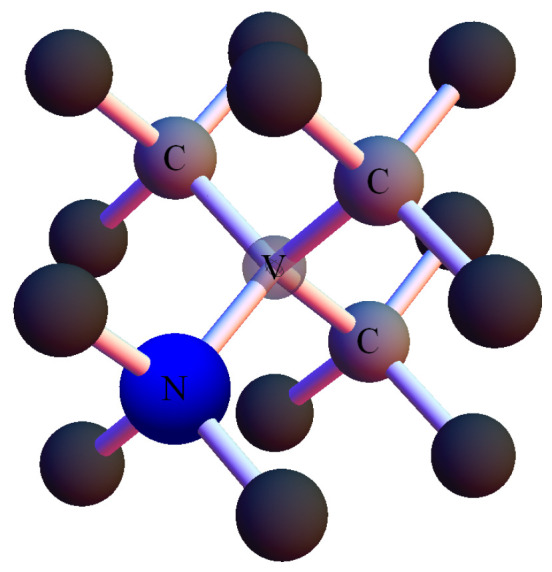
Single NV center in a diamond crystal carbon lattice unit cell.

The nitrogen-vacancy (NV) center in diamond (point-like defects in a carbon lattice) is a robust, optically addressable quantum system exhibiting spin-dependent fluorescence, single-photon emission and long coherence times at room temperature [10]. For an NV center, the vacancy (V) is surrounded by three carbon atoms and a carbon atom-substituting nitrogen atom (N) that can be located in one of the four possible positions (directions); see Figure 1. Diamond is a compact, physically and chemically robust as well as non-toxic platform, capable of operating in harsh environments with high pressure of up to 60 GPa and temperatures ranging from cryogenic temperatures to 600 K [11]. In this work we investigate both ensemble and single-emitter NV-based QRNG implementations and analyze their statistical performance.

NV centers in diamond have been used for QRNG in previous studies as single-photon sources also in combination with a beamsplitter, achieving about 34 randomly generated bits per second [12]. In this study we quantify the nanostructure effect on the quality of the generated bit randomness.

## 2. Principles of Randomness

We looked at two randomness extraction methods in this study: a time-of-arrival method [13] and a beamsplitter method [14].

The time-of-arrival method allows us to create random bits by separating our data in discrete bins. We choose a set time bin width Δt, which will be used to extract random bits from the time of arrival data. Once the time scale has been discretized, we need to check if a photon detection event has occurred. If a single or many events occurred in the chosen interval then we set that bin’s value to “1”, and if no events occurred, to “0”. By doing this, we get a random binary sequence. The width of the time bin comes from Poisson distribution (1) when choosing the highest variation.

(1) $$P=1-e^{-\lambda \Delta t}.$$

The value λ is defined as the total events per time interval and can be calculated as the total photon count *N* over the full time scale *t* (in the case of the data presented here, λ varies depending on the red photon flux, on the order of tens of kilo-counts per second).

(2) λ=N/t

The width we can define as Δt=n/λ, where *n* is the expected counts per bin. Since we want to get the highest order of variance, we set n=ln(2), from which we will get P=0.5. Since both λ and *n* are known, we can calculate the optimal Δt. Since the parameter λ dynamically changes per data set, we cannot choose one single bin width. In most cases the average bin width for the measured data sets was determined to be 45 μs (optimizing for the lowest bias in the results). As the binning aggregates detection events over finite time intervals, it is possible that it can induce correlations due to detector dead time, afterpulsing, or slow system fluctuations (we did not evaluate whether the binning procedure introduced temporal correlations in the generated bit sequences in the scope of this study).

Quantum mechanics guarantees that when a photon hits a balanced beamsplitter, the outcome—the photon is transmitted or reflected—is intrinsically random. No hidden variables or classical mechanisms determine the result. This physical process gives a natural 1-bit random variable: detector A clicks—output 0; detector B clicks—output 1. Quantum mechanics describes the output state as a superposition:

(3) $$|\psi_{\mathrm{out}}\rangle =\frac{1}{\sqrt{2}}(|\mathrm{transmitted}\rangle +|\mathrm{reflected}\rangle ).$$

This superposition collapses only when the photon is detected. Because quantum mechanics dictates that the photon cannot “decide” the path beforehand, each bit is fundamentally unpredictable.

## 3. Experimental Setup

**Figure 2:**
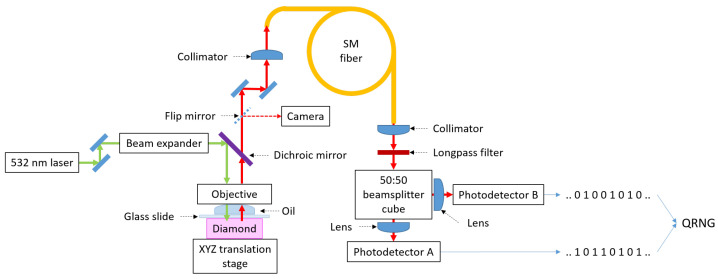
Experimental setup.

We constructed a table-top confocal microscope experimental setup for locating single NV centers in diamonds (see Figure 2)—a device prototype for quantum random number generation. In a confocal system, both a physical pinhole and a single-mode optical fiber can act as the spatial filter that improves Z-resolution. We used a single-mode optical fiber [15] instead of a pinhole [16] for confocal microscopy due to the fact that a pinhole is more prone to mechanical drift sensitivity, slight misalignment degrades resolution, and it does not spatially filter mode structure. A single-mode fiber provides better spatial filtering (higher contrast), excellent background rejection, improved signal stability and mode cleaning. The output from the optical fiber is sent to a Hanbury Brown–Twiss setup—a 50:50 beamsplitter [17].

To excite the NV centers in diamond, a 532 nm green laser (Verdi V18 by Coherent)) was operated at 1.0 W output power with a plate polarizer (PBSW-532 by Thorlabs) placed in the path of the laser beam at a slight angle to divert away any back reflections from the laser output aperture coming from the rest of the experimental setup, and the laser power after the plate polarizer was 10 mW. Free-space mirrors were used to guide the light to the diamond to avoid any laser power fluctuation noise introduced by optical fibers. The guided green light was focused on the diamond through an objective (Plan Fluor 100x/1.30 Oil WD 0.16 by Nikon). A beam expander (GBE03-A by Thorlabs) was used to backfill the objective (for increased resolution). Immersion oil (type LDF, formula code 387, by Cargille Laboratories) was used in combination with a glass slide.

The emitted red fluorescence from the diamond was gathered through the same objective. A dichroic mirror (DMLP605 by Thorlabs), a single-mode optical fiber and a longpass filter (FELH0550 by Thorlabs) were used to separate the emitted red fluorescence from the green exciting light and guide it to two single-photon detectors (SPDMH2 by Thorlabs; active area diameter: 100 μm; photon detection efficiency: 70% at 670 nm; dead time between detected photons: 45 ns; afterpulsing probability: 0.2%) through a 50:50 beamsplitter cube (CCM1-BS013/M by Thorlabs). We used collimators to guide the light in the single-mode fiber (TC25APC-633 by Thorlabs) and to collect the light coming out from the fiber (F240FC-532 by Thorlabs). The TTL signals from the single-photon detectors were synchronized and collected using an oscilloscope (RTB2004 by Rohde & Schwarz; using a 200 μs per division time-scale resolution). The diamond was moved with respect to the objective using a XYZ translation stage (NanoMax-TS MAX373DK1/M by Thorlabs).

The objective in combination with the immersion oil, when back filled using the beam expander, yields a XY resolution (diffraction-limited, ideal case) of about 300 nm for red 650 nm light, so we set our measurement step to be 0.25 μm for moving in the X and Y directions with the translation stage.

### 3.1. Nanodiamond Ensemble QRNG

**Figure 3:**
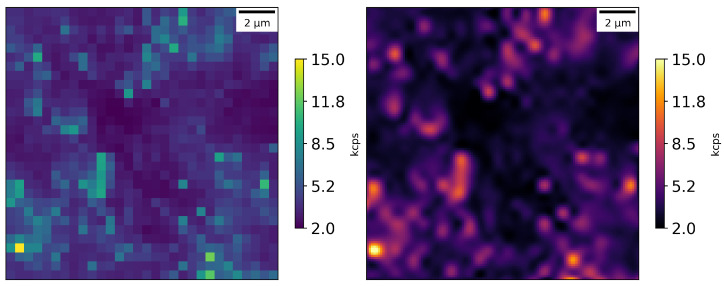
Photon counts from nanodiamonds in an area of 15 × 15 μm: raw data on left and cubic interpolation on the right; scan step size—0.25 μm.

We used 100 nm scale fluorescent nanodiamonds (FNDs) with an NV center concentration of approximately 3 ppm. Typically, for this type of nanodiamonds, the number of NV centers per nanocrystal are on the order of 100. The nanodiamonds were used to firstly calibrate our confocal microscope setup, optimizing for red fluorescence collection efficiency and data collection rate; see Figure 3. The experimental setup can distinguish signals from NV centers with a contrast of around 90% with respect to the background. The nanodiamonds were prepared by mixing, diluting them in isopropanol (using sonication) and depositing a small drop of this solution on a microscope glass slide.

In nanodiamond crystals containing randomly distributed NV centers, spontaneous emission timing is governed by quantum decay processes. Photon detection time fluctuations are fundamentally quantum. Random bits are extracted from photon arrival time bins as explained above.

### 3.2. Single NV Nanopillar QRNG

**Figure 4:**
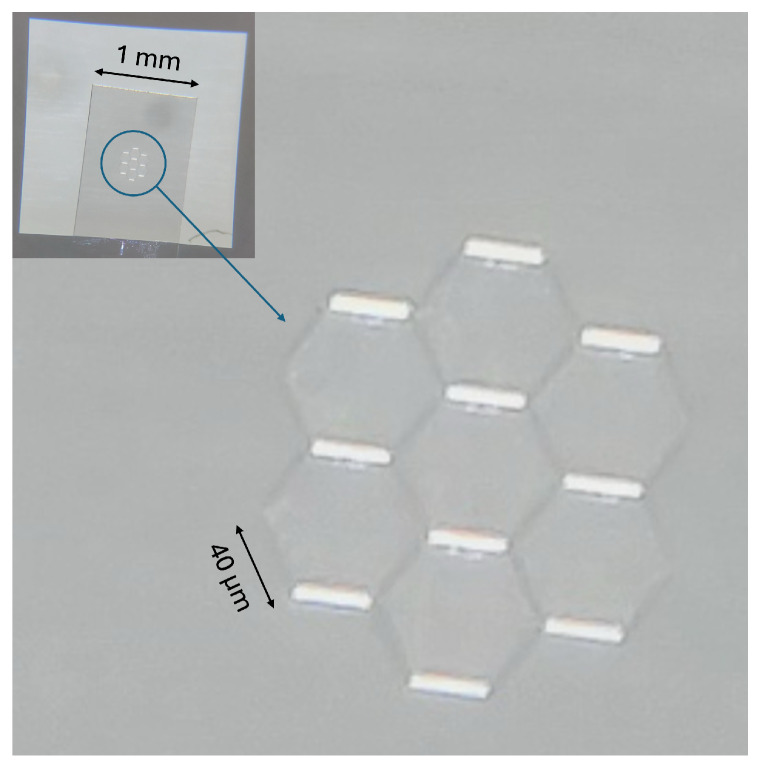
Microscope image of the diamond sample with fabricated nanopillar structures with NV centers inside a protective hexagonal pattern; 4x magnification, where one side of an individual hexagon is 40 μm long and the spacing between individual nanopillars is 2 μm.

We also used a diamond sample with single NV centers located at the top of fabricated nanopillars on a diamond surface from a company—Qnami, Muttenz, Switzerland; see Figure 4. The manufacturer states that in 30% of cases, there is a single NV center located at the tip of a single nanopillar (a few NV centers or none in other cases).

**Figure 5:**
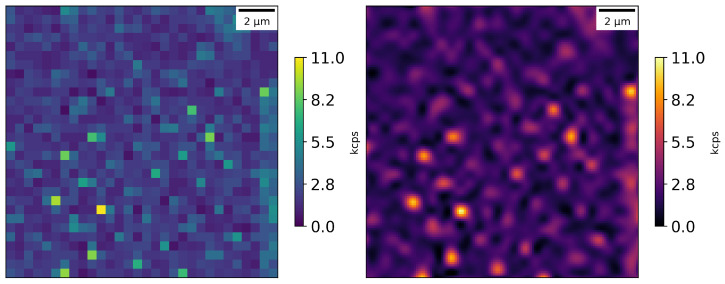
Photon counts from the nanopillar diamond in an area of 15 × 15 μm: raw data on the left and cubic interpolation on the right; scan step size—0.25 μm.

These types of nanopillars improve the light collection efficiency by about 20 times compared with typical bulk diamonds, yielding higher signal-to-background ratios; see Figure 5. It can be seen that with our experimental setup configuration, we are detecting approximately 28 times fewer counts from the nanopillar diamond than from the nanodiamonds (many NV centers in brighter spots, approximately 3 ppm), but signals from the nanopillars are still clearly visible (few or single NV centers at each bright spot).

Single NV centers ensure emission of one photon at a time. Randomness arises from beamsplitter path selection (50:50). This approach provides higher entropy certification since it is based on single-quantum events.

## 4. Results

Here we present the analysis of the generated random numbers from both measurement cases—nanodiamonds and single NV centers at nanopillars. We calculated the conditional min-entropy and performed NIST SP 800-22 tests [18].

The quality of the generated random bit sequences is quantified using conditional min-entropy, a standard metric in cryptographic randomness certification. For a discrete random variable *X* with outcomes *x*, the min-entropy is defined as $H_{min}=-{\text{log}}_{2}\;\left(max_{x}p\left(x\right)\right)$, reflecting the probability of the most likely outcome—that is, the success probability of an optimal single-guess attack on the output. Unlike Shannon entropy, which measures average uncertainty, min-entropy provides a conservative worst-case bound particularly suited to security applications. A value of $H_{min}=1$ bit corresponds to a perfectly uniform binary source in which both outcomes are equally likely and no prediction strategy performs better than random guessing, while $H_{min}=0$ indicates a fully deterministic source. The conditional min-entropy used here, defined in Equation (4), extends this to account for any piece of side information *y* available to an adversary, and is estimated from the measured detector click probabilities $P_{A}$ and $P_{B}$ following the approach in [12]:

(4) $$H_{\text{min}}=-{\text{log}}_{2}\;\left(\sum_{y}p\left(y\right)\;\underset{x}{\text{max}}p\left(x|y\right)\right).$$

### 4.1. Results Using NV Centers in Nanodiamonds

**Figure 6:**
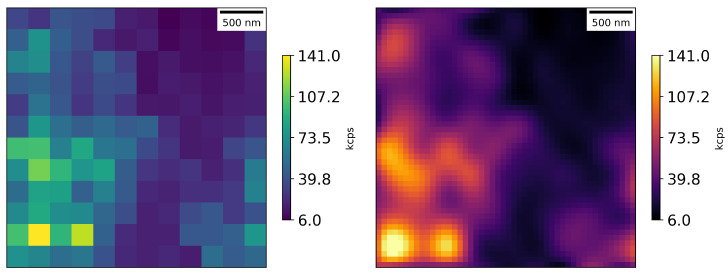
Photon counts from the nanodiamonds in an area of 3 × 3 μm: raw data on the left and cubic interpolation on the right; scan step size—0.25 μm.

Focusing specifically on a few nanodiamonds we get a photon flux on the order of 140 kcps for the brightest spot, see Figure 6.

Results of NIST SP 800-22 tests for the brightest nanodiamond spot are compiled in Table 1. The original values for generating random bits using the time binning method described above without detector debiasing, i.e., detector A and B click probabilities, are $P_{A}$ = 0.2130 and $P_{B}$ = 0.7870; total counts = 31,998. The calculated conditional min-entropy is $H_{\min}$ = 0.3455 bits.

Due to the fact that the detectors are not perfectly even and the light going in to both detectors is not perfectly aligned, we used the Von Neumann debiasing [19] method for the registered photons. After debiasing: $P_{A}$ = 0.5001, and $P_{B}$ = 0.4999; total counts = 4025. $H_{\min}$ = 0.9786 bits. It can be seen that the min-entropy for the debiased data indicates higher quality of randomness for the data at the cost of reduced bit count—31,998/4025 = 7.9 times less bits.

Von Neumann debiasing was selected due to its implementation simplicity and its provably secure, assumption-free output. It requires no knowledge of the bias structure and produces output bits that are exactly uniform without introducing free parameters that could complicate interpretation across the two sources. Its principal drawback is efficiency, since for a source with bias *p*, the output rate scales as 2p(1−p), approaching zero for strongly biased inputs.

More efficient alternatives that could be used for improved implementations exist. The Peres extractor [20], a recursive generalization of Von Neumann debiasing, processes discarded bit pairs to approach the entropy rate while retaining the seedless, deterministic character of Von Neumann debiasing. Toeplitz matrix hashing [12,21], a construction from the two-universal hash family, achieves near-optimal extraction close to the min-entropy bound and only requires a valid lower bound on min-entropy—making it theoretically better suited to single-photon sources exhibiting antibunching. Both represent well-established progressions toward a certified, high-throughput implementation and are the recommended post-processing methods for a production QRNG system based on the architectures demonstrated here.

### 4.2. Results Using a Single NV Center in a Structured Diamond with Nanopillars

**Figure 7:**
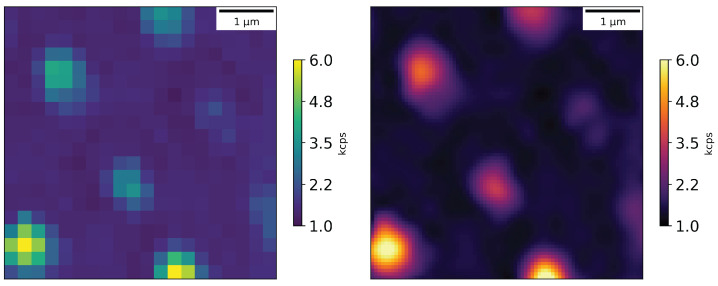
Photon counts from the nanopillar diamond in an area of 5 × 5 μm; raw data on the left and cubic interpolation on the right; scan step size—0.25 μm.

Focusing on one particular bright spot in the nanopillar diamond (see Figure 7) we get a photon flux on the order of 5 kcps.

Results of NIST SP 800-22 tests for one bright nanopillar diamond spot are compiled in Table 2.

The original values, without detector debiasing, of detector A and B click probabilities are $P_{A}$ = 0.4319 and $P_{B}$ = 0.5681; total counts = 19,276. The calculated conditional min-entropy is $H_{\min}$ = 0.8158 bits. After debiasing, these are $P_{A}$ = 0.4941 and $P_{B}$ = 0.5059; total counts = 5015. $H_{\min}$ = 0.9817 bits, with a reduced bit count of 19276/5015 = 3.8 times less bits.

## 5. Discussion

The two QRNG implementations investigated in this work operate in fundamentally different physical regimes. The nanodiamond system consists of an ensemble of NV centers, producing multi-emitter fluorescence with effectively Poissonian photon statistics, whereas the nanopillar device isolates individual or few NV centers and approaches the single-emitter regime. This distinction directly impacts the intrinsic randomness properties of the generated bit streams. Their photon statistics and noise characteristics differ, and a direct comparison of randomness metrics such as min-entropy does not isolate material or device performance. A controlled comparison would require normalization of photon statistics and emitter number. The comparison presented here is phenomenological, rather than a strict equivalent comparison. The observed differences in entropy may arise from both photon statistics (multi-photon vs. single-photon emission) and experimental factors, such as signal-to-noise ratio, detector response and experimental setup throughput.

The nanodiamond ensemble exhibits significantly higher brightness, with count rates approximately 28 times larger than those observed for the nanopillar structures. This is primarily due to the presence of multiple emitters within each nanocrystal and a larger effective excitation volume. In contrast, the nanopillar count rate is limited by the excited-state lifetime of a single NV center (10–20 ns), resulting in lower photon flux but improved control over emission statistics.

This difference in photon statistics is reflected in the raw entropy values. Prior to any post-processing, the nanodiamond system exhibits substantial bias ($H_{\min}=0.3455$), arising from multiple emitters, larger excitation volume, background fluorescence, and unequal coupling into the detection channels. While multi-photon events in these ensembles can be partially filtered to improve statistics, it occurs at the cost of further decreasing extraction efficiency. The nanopillar system shows significantly higher intrinsic min-entropy ($H_{\min}=0.8158$), consistent with reduced multi-photon contributions and more balanced detection probabilities.

After applying Von Neumann debiasing [19], both systems converge to high-quality randomness with min-entropy values close to unity (0.98 bits). However, this comes at the cost of reduced bit rates. The nanodiamond implementation requires approximately an eightfold reduction in data, while the nanopillar system requires only a fourfold reduction. This demonstrates that the nanopillar approach provides higher entropy efficiency, as a larger fraction of the raw data can be retained after bias removal.

Von Neumann debiasing removes first-order bias but does not eliminate higher-order correlations or memory effects. Therefore, the quality of the initial physical randomness source remains critical. The improved performance of the nanopillar system reflects its more favorable photon statistics rather than post-processing alone.

The statistical testing results further reflect limitations imposed by finite data size. Several NIST SP 800-22 tests are marked as “ineligible” due to insufficient bit sequence length relative to the requirements (at least $10^{6}$ bits) of the test suite [18]. In addition, failures in tests such as the Random Excursion test are attributed to statistical fluctuations arising from limited data rather than systematic deviations from randomness. The tests that could be applied consistently show no statistically significant departure from random behavior. All eligible tests pass with *p*-values below the significance thresholds for both systems. Extending data sets for full NIST suite validation should be the direct next step toward a fully certified implementation.

Although single NV centers are expected to exhibit photon antibunching [22], a quantitative measurement of the second-order autocorrelation function $g^{\left(2\right)}\left(0\right)$ was limited by insufficient coincidence statistics with the present experimental setup implementation. A precise determination of $g^{\left(2\right)}\left(0\right)$ would require longer acquisition times and improved experimental setup throughput.

Compared with QRNG systems based on internal noise sources such as vacuum fluctuations or laser phase noise [8], which can achieve Mbps to Gbps bit rates, NV center-based QRNG systems operate at significantly lower rates but provide a more direct physical link between the quantum emission process and the generated randomness. This enables more transparent entropy estimation and source characterization, at the cost of reduced throughput.

Nanopillar design optimizations have primarily focused on enhancing photon collection efficiency, improving ODMR contrast, and increasing single-photon source brightness [23]. Further optimization specifically targeting QRNG performance, such as maximizing emission rate while minimizing background contributions, represents an important direction for future work.

## 6. Conclusions

We have demonstrated and compared two solid-state quantum random number generation implementations based on NV centers in diamond: an ensemble nanodiamond system and a nanopillar-isolated single-emitter system, both operating at room temperature.

The results reveal a clear trade-off between photon flux and intrinsic randomness quality. Nanodiamond ensembles provide higher count rates (raw bits) but exhibit significant bias due to multi-emitter fluorescence and background contributions, requiring substantial post-processing. In contrast, nanopillar-isolated NV centers exhibit higher intrinsic min-entropy (randomness quality), reflecting more favorable photon statistics.

After debiasing, both systems achieve high-quality randomness with min-entropy values approaching unity. However, the nanopillar approach demonstrates higher entropy efficiency, retaining a larger fraction of usable bits.

Diamond-based QRNG platforms provide a robust and potentially scalable route toward randomness generation with room-temperature operation and compatibility with integrated quantum technologies.

## Figures and Tables

**Table 1:** NIST test results (after debiasing) for nanodiamonds.

Test Name	Passed	*p*-Values
Monobit	True	0.9874
Frequency Within Block	True	0.8044
Runs	True	0.3524
Longest Run Ones In A Block	True	0.5747
Binary Matrix Rank	Ineligible	
Discrete Fourier Transform	True	0.8395
Non-Overlapping Template Matching	True	0.9894
Overlapping Template Matching	Ineligible	
Maurers Universal	Ineligible	
Linear Complexity	Ineligible	
Serial	True	0.4143–0.5155
Approximate Entropy	True	0.2914
Cumulative Sums	True	0.9779, 0.9724
Random Excursion	False	0–0.2436
Random Excursion Variant	True	0.0105–0.8382

**Table 2:** NIST test results (after debiasing) for nanopillar diamond.

Test Name	Passed	*p*-Values
Monobit	True	0.4048
Frequency Within Block	True	0.2913
Runs	True	0.3789
Longest Run Ones In A Block	True	0.2238
Binary Matrix Rank	Ineligible	
Discrete Fourier Transform	True	0.2623
Non-Overlapping Template Matching	True	0.9990
Overlapping Template Matching	Ineligible	
Maurers Universal	Ineligible	
Linear Complexity	Ineligible	
Serial	True	0.3441–0.5038
Approximate Entropy	True	0.5819
Cumulative Sums	True	0.2475–0.7056
Random Excursion	False	0–0.7674
Random Excursion Variant	False	0.0–0.8839

## Data Availability

The original contributions presented in this study are included in the article. Further inquiries can be directed to the corresponding authors.
